# Fermentation-Driven Valorization of a Carrot Juice By-Product into an Exopolysaccharide-Enriched Beverage

**DOI:** 10.3390/foods15030451

**Published:** 2026-01-27

**Authors:** Mario Caponio, Lorenza Francesca De Lellis, Maria Daglia, Michela Verni, Carlo Giuseppe Rizzello

**Affiliations:** 1 Department of Soil, Plant, and Food Sciences, University of Bari, 70125 Bari, Italy; mario.caponio@uniba.it; 2 Department of Pharmacy, University of Naples Federico II, 80131 Naples, Italy; lo.delellis2@gmail.com (L.F.D.L.); maria.daglia@unina.it (M.D.); 3 International Research Center for Food Nutrition and Safety, Jiangsu University, Zhenjiang 212013, China; 4 Department of Environmental Biology, Sapienza Università di Roma, 00185 Rome, Italy; carlogiuseppe.rizzello@uniroma1.it; 5 Department of Human Science and Quality of Life Promotion, San Raffaele University, 00166 Rome, Italy

**Keywords:** carrot by-products, fermentation, exopolysaccharides, functional beverages, circular bioeconomy

## Abstract

Carrot juice processing generates large amounts of pomace, a fibre-rich by-product with significant valorisation potential. This study explored the feasibility of fermenting carrot by-product with *Levilactobacillus brevis* AM7 and *Leuconostoc pseudomesenteroides* DSM20193 to produce exopolysaccharide (EPS)-enriched functional beverages. Beverages were fermented with or without sucrose addition (EPS^+^ and EPS^−^, respectively) and characterized for microbiological, biochemical, rheological, and sensory attributes. Both strains showed robust growth (>8 log cfu/mL) and acidification (final pH below 4.8), comparable to plant-based yoghurt alternatives, with EPS synthesis markedly enhanced in sucrose-supplemented beverages. *Leuc. pseudomesenteroides* DSM20193 synthesized the highest EPS concentration (16.8 g/100 g dry weight), resulting in a 6-fold viscosity increase compared to EPS^−^ samples, thus improving the adherence to the spoon and preventing syneresis of the beverages. Sensory evaluation revealed that EPS^+^ carrot-based beverages had improved sweetness due to a slight sucrose residue, aroma, and mouthfeel, while maintaining low off-flavours and high colour uniformity. The results highlight carrot by-product as a promising substrate for developing clean-label beverages that are rich in dietary fibres and polyphenols and show antioxidant and potential prebiotic properties through sustainable fermentation processes.

## 1. Introduction

Carrot (*Daucus carota* L.) is one of the world’s most widely cultivated root vegetables and a key ingredient in the juice processing industry [1]. During industrial carrot juice extraction, a substantial portion of the original raw material is separated as a wet solid by-product, which includes peelings, cortical tissue, vascular elements and insoluble fractions of the carrot matrix [2]. Such a by-product, commonly referred to as carrot pomace, press cake or carrot residue, is rich in dietary fibre, comprising cellulose, hemicellulose, pectin and some lignin; besides containing carbohydrates, carotenoids (notably β- and α-carotene), polyphenols and minor amounts of proteins and lipids. This indeed makes it an attractive substrate for biorefinery and food applications, provided appropriate processing and stabilization are applied [2,3].

Several studies point to the magnitude of this side stream, with global carrot pomace production estimated to be on the order of tens of millions of tonnes per year. This volumetric scale underlines the need for economically viable valorisation routes because transportation, storage and disposal of high-moisture residues are costly and environmentally impactful [2,3,4]. Depending on local economics, legislation, proximity to animal farms, availability of composting or anaerobic digestion facilities and market demand for by-product-derived ingredients, industrial and small-scale processors employ several utilisation options for carrot pomace [2,5]. Major disposal routes include direct feed and feed ingredient [6], disposal in landfills [2], anaerobic digestion for biogas production or incineration [5], as well as direct use as a food ingredient. Indeed, some studies have evaluated the possibility of using carrot residue to produce high-fibre bread, cakes and biscuits [7,8]. However, deterioration in the organoleptic and structural characteristics of the products is often observed at high levels of inclusion, making it necessary to reduce the percentage of addition, which consequently limits the potential beneficial effect [7].

Therefore, repurposing strategies within a circular economy framework should be focused on the design of novel food products that balance sustainability goals with technological feasibility and consumer acceptability. In this context, fermentation represents a promising biotechnological tool to enhance both the functional and sensory characteristics of carrot-based matrices, since several lactic acid bacteria (LAB) are able to synthesize a broad group of high-molecular-weight molecules, like exopolysaccharides (EPS) [9,10]. EPS are composed of monosaccharide units linked by glycosidic bonds, which exhibit a variety of structure-functional properties and biological activities [9,11]. Even though most investigations deal with the application of EPS in the food industry for structural reasons, having emulsifying, texturizing and water-binding properties, their health-promoting potential has also been demonstrated, including immunomodulatory, prebiotic, anti-inflammatory, anti-biofilm and antioxidant activities [10,12].

However, the literature on carrot pomace fermentation is rather scarce. Indeed, while fermentation of carrot pomace was used to produce bioethanol or organic acids [13], reutilization strategies within the food industry after fermentation are limited. These include fermentation with *Lactobacillus casei* to improve its antioxidant potential [14] or with *Saccharomyces cerevisiae* to release antimicrobial polyphenols active against *Escherichia coli* [15]. Still, no research has ever focused on the suitability of carrot pomace as a fermentation substrate for a beverage enriched in EPS. Hence, based on the above considerations, the present study sought to evaluate the technical feasibility of using carrot residue derived from carrot juice extraction as a fermentation substrate for selected lactic acid bacteria. EPS-enriched beverages were produced using carrot pomace as sole ingredient, and the main microbiological and biochemical features evaluated. EPS were also quantified, and the beverages were characterized for their main organoleptic properties.

## 2. Materials and Methods

### 2.1. Raw Materials and Microorganisms

For this study, carrot by-product, kindly provided in the form of pellets by Aureli Mario S.S. Agricola (Ortucchio, AQ, Italy), was used. The carrot-derived pellets had a moisture of 5% and the following composition: dietary fibre 58%; carbohydrates 24%; protein 7%; ash 5%; and lipid 1%.

Two LAB strains, *Levilactobacillus brevis* AM7 and *Leuconostoc pseudomesenteroides* DSM20193, selected for the ability to produce exopolysaccharides and previously used for the fermentation of vegetal matrices [16,17,18], belonging to the Culture Collection of the Department of Environmental Biology (Sapienza University of Rome), were used as starters. Strains were routinely propagated in De Man, Rogosa and Sharp (MRS) (Oxoid, Basingstoke, Hampshire, UK) broth at 30 °C for 24 h. Before use, cells were harvested by centrifugation at 10,000 rpm at 4 °C for 10 min and washed twice in sterile 0.9% NaCl (pH 7.0) and resuspended in tap water.

### 2.2. Carrot By-Product Fermentation

For the fermentation, carrot-derived dried pellets were suspended in tap water at 2.5% (*w*/*v*), allowed to rehydrate for 30 min and homogenized with a blender (Cecotec, Valencia, Spain) to obtain a smooth suspension. Then cells, collected as previously described, were singly inoculated at a cell density of approx. 6.5 log cfu/g. A total of four beverages were produced using *Lv. brevis* AM7 and *Leuc. pseudomesenteroides* DSM20193 as a starter for the fermentation, obtaining AM7 EPS^−^ and DSM EPS^−^, respectively, and AM7 EPS^+^ and DSM EPS^+^ when a 5% (*w*/*v*) sucrose supplementation was performed. Fermentation was carried out at 25 °C for 24 h. A not-inoculated beverage (Ct) was prepared in the same conditions and used as a control (Table 1).

### 2.3. Microbiological and Biochemical Characterization

For the microbiological analysis, 10 g of each sample were diluted with 90 mL of physiological solution (0.9%), homogenized with a Stomacher Lab-Blender 400 (Seward Medical, London, UK) for 3 min and subjected to serial decimal dilutions. LAB, yeasts and *Enterobacteriaceae* were respectively determined on MRS, Saboraud and Violet Red Bile Glucose Agar (Oxoid).

The proximate composition of the beverages (proteins, lipids, moisture, total dietary fibre, and ash) was determined according to the Approved Methods of the American Association of Cereal Chemists 46–11.02, 30–10.01, 44–01.01, 32–05.01, and 08–01.01 [19].

The pH was monitored with a FiveEasy Plus pH metre (Mettler-Toledo, Columbus, OH, USA), whereas total titratable acidity (TTA) was determined on 10 g of product homogenized with 90 mL of distilled water and expressed as a quantity (mL) of 0.1 M NaOH needed to reach a pH of 8.3.

The analysis of organic acids and sugar was carried out on the supernatant resulting from centrifugation of the beverages at 12,000 rpm for 20 min. The determination of lactic acid and acetic acid was carried out by using the Megazyme K-DLATE and K-ACETRM kits (Megazyme International, Wicklow, Ireland), respectively, following the manufacturer’s instructions. The fermentation quotient was calculated as the molar ratio between lactic and acetic acid. Glucose, fructose and sucrose were determined by using the Megazyme K-FRUGL and K-MASUG kits (Megazyme International) following the manufacturer’s instructions.

The total polyphenol content (TPC) of the samples was determined using the Folin–Ciocalteu method, with slight modifications [20]. Specifically, 50 mg of the freeze-dried samples were solubilized into 1 mL of ethanol-water solution (1:1 *v*/*v*). Then, a 10 µL aliquot of each sample was mixed with 50 µL of Folin–Ciocalteu reagent. Gallic acid was used to create a 9-point calibration curve, ranging from 200 to 1000 µg/mL. The R^2^ value of the regression equation was higher than 0.99. After cyclomixing for 4 min, 200 µL of 15% Na_2_CO_3_ were added. Distilled water was added to reach a final volume of 1 mL. Samples were incubated for 2 h at room temperature, and absorbance was measured at 750 nm. TPC was expressed as mg gallic acid equivalents per gram of sample (mg GAE/g, dry weight basis).

The antioxidant potential of the beverages was determined as radical scavenging activity on 1,1-diphenyl-2-picrylhydrazyl (DPPH) free radical [21]. In detail, sample extracts obtained as reported above were added to 2 mL of 0.1 mM DPPH dissolved in 95% ethanol. The mixture was shaken and left for 30 min at room temperature, and the absorbance of the resulting solution was read at 517 nm. The scavenging effect was expressed as follows:

$$\mathrm{DPPH}\;\mathrm{scavenging}\;\mathrm{activity}\;\left(\%\right)=\;\frac{\left(\mathrm{blank}\;\mathrm{absorbance}\;-\;\mathrm{sample}\;\mathrm{absorbance}\right)}{\mathrm{blank}\;\mathrm{absorbance}}\;\times \;100$$

Butylated hydroxytoluene (BHT) was assayed as an antioxidant reference (75 ppm).

### 2.4. EPS Isolation and Quantification and Viscosity Analysis

EPS were isolated and quantified on 5 g of sample using the gravimetric method described in Ketabi et al. [22]. Briefly, the beverages underwent protein precipitation with trichloroacetic acid (20%); then, EPS were precipitated with ethanol, recovered by centrifugation, and weighed after freeze-drying in a Lyovapor™ L-210 (Buchi Ltd., Newmarket, Suffolk, UK).

Viscosity was determined by using a rotational viscometer Lch. NDJ-8S (Lachoi Scientific Instrument Co., Shaoxing, China). The viscosity measurement was carried out on 50 g samples, placed in beakers having a 7 cm diameter and 12.5 cm height, at 25 °C, by using the speed/rotor combinations suggested by the viscometer provider, to fall within the correct calibration ranges of the instrument. More specifically, the optimized conditions were obtained using the spindle n. 2 provided by the manufacturer at 3 rpm.

### 2.5. Colour and Sensory Analysis

The chromaticity coordinates of the beverages were obtained by a CS-10 colourimeter (CHN Spec Technology, Hangzhou, China) and reported as color difference, Δ*E* ∗ *ab*, calculated by the following equation:

$$\Delta E*ab=\sqrt{{\left(\Delta L\right)}^{2}+{\left(\Delta a\right)}^{2}+{\left(\Delta b\right)}^{2}}$$

where Δ*L*, Δ*a* and Δ*b* are the differences for *L*, *a* and *b* values between sample and reference (a white ceramic plate having *L* = 92.2, *a* = 0.15, and *b* = 0.85).

Preliminary sensory analyses were carried out by 10 trained panellists (4 men and 6 women; average age: 32 years; range: 24–45 years) with demonstrated abilities and prior expertise in plant-based product assessment. A two-hour training session was performed, and the assessors evaluated the descriptors to be included in the sessions. Sensory attributes were scored on a scale from 0 to 10, with 10 being the highest score. Sensory evaluations were carried out following the independent method of the “Sensory analysis—Methodology—Flavour Profile” methods (ISO 6564-1985) with some modifications. In detail, the library of the Environmental Biology Department of the Sapienza University of Rome (Italy) was used instead of cabinets as previously proposed by Elia [23]. Enrolled panellists, who did not suffer from any food intolerances or allergies, received information on the objectives of this study and provided written informed consent. Three separate sessions were conducted, and beverages were served in a randomized order and encoded with three-digit random numbers. A glass of water was drunk by the panellists between samples.

### 2.6. Statistical Analysis

Analyses were carried out on samples obtained in three separate replicates, and each sample was analysed in duplicate. Data were subjected to one-way ANOVA; paired comparisons of treatment means were achieved by Tukey’s procedure at *p* < 0.05, using the software Statistica 12.5 (StatSoft Inc., Tulsa, OK, USA).

## 3. Results

### 3.1. Proximal Composition

The proximal composition of the carrot-based beverages highlighted differences only in carbohydrate content (Table 2). Indeed, soluble sugars were significantly higher, up to 3-fold, in AM7 EPS^+^ and DSM EPS^+^, compared to the control beverage. Dietary fibre, protein, fat and ash contents did not significantly differ (*p* > 0.05) among samples, maintaining values around 1.5, 0.15 and 0.04 g/100 g for dietary fibres, proteins and fats, respectively.

### 3.2. Microbial Growth

**Figure 1:**
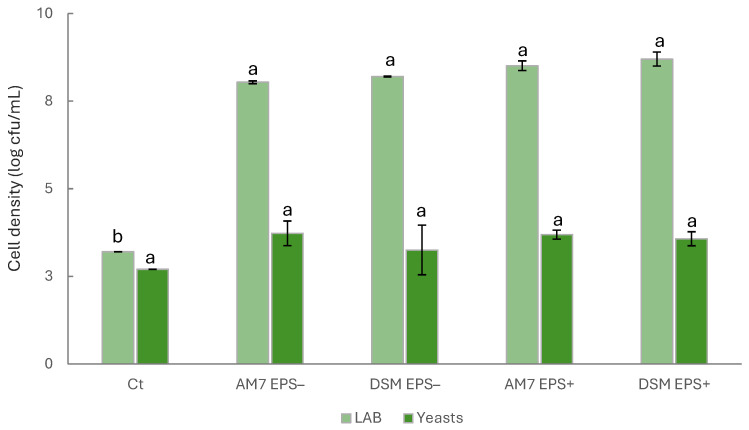
Cell density, expressed as log cfu/g, of lactic acid bacteria (LAB) and yeasts of carrot-based beverages before (Ct) and after 24 h of fermentation at 25 °C with *Leuc. pseudomesenteroides* DSM20193 (DSM) and *Lv. brevis* AM7 (AM7), with (EPS^+^) or without added sucrose (EPS^−^). ^a,b^ Values with different superscript letters, within the same parameter, mean significant differences at *p* < 0.05.

The main microbial groups in the beverages were enumerated before and after fermentation (Figure 1). The cell density of presumptive LAB was 3.2 ± 0.01 log cfu/mL before the inoculum (Ct). After fermentation, it increased by approximately 2 log cycles, exceeding 8 log cfu/mL, regardless of the strain used. Although slightly higher (*p* > 0.05), LAB growth did not differ (*p* > 0.05) between sucrose-containing samples (EPS^+^) and EPS^−^ samples. Yeasts, on the other hand, showed an initial density of 2.7 ± 0.01 log cfu/mL in Ct and remained stable around 3 log cfu/mL during the incubation of all samples. Similarly, *Enterobacteriaceae* remained below 10 cfu/mL in all the beverages, either before or after fermentation.

### 3.3. Acidification, Carbohydrate Metabolism and Antioxidant Potential

Fermentation led to a marked acidification of the beverages; indeed, the initial pH and TTA of the beverages were ca. 5.9 and 0.7, respectively. After fermentation with *Lv. brevis* AM7 and *Leuc. pseudomesenteroides* DSM20193, compared to the control, significant decreases in pH were observed. Correspondingly, TTA increased, reaching the highest and lowest values in AM7 EPS^+^ and DSM EPS^−^, respectively.

Carbohydrate metabolism varied among treatments. Glucose, the least abundant sugar in the matrix, significantly decreased (by more than 40%) after fermentation, particularly in AM7 EPS^+^. Endogenous sucrose, which had the highest concentration among sugars (roughly 10 and 5 times higher than glucose and fructose, respectively) was also found in fermented samples. Nevertheless, residual sucrose levels were markedly higher (*p* < 0.05) after EPS^+^ fermentation and decreased in EPS^−^ samples compared to Ct. As a hydrolysis product of sucrose, fructose was found in concentrations notably higher than the control only in beverages fermented with *Leuc. pseudomesenteroides* DSM20193 (Table 3).

Before fermentation, the beverages contained negligible amounts of organic acids, whereas in fermented drinks, lactic and acetic acids ranged from 2.4 to 3.3 and from 0.2 to 1.1 mmol/kg, respectively, depending on the sample (Table 3). Consequently, the fermentation quotient of the beverages was 3.9, 20.4, 2.4 and 2.6 for AM7 EPS^−^, AM7 EPS^+^, DSM EPS^−^ and DSM EPS^+^, respectively.

Fermentation also led to a release of phenolic compounds; indeed, TPC significantly increased (*p* < 0.05) (from 13 to 66%), with AM7 EPS^+^ and AM7 EPS^−^ showing the highest and lowest values, respectively. Similarly, DPPH radical scavenging activity was higher in fermented beverages compared to the control (Table 3).

### 3.4. EPS Synthesis and Viscosity

**Figure 2:**
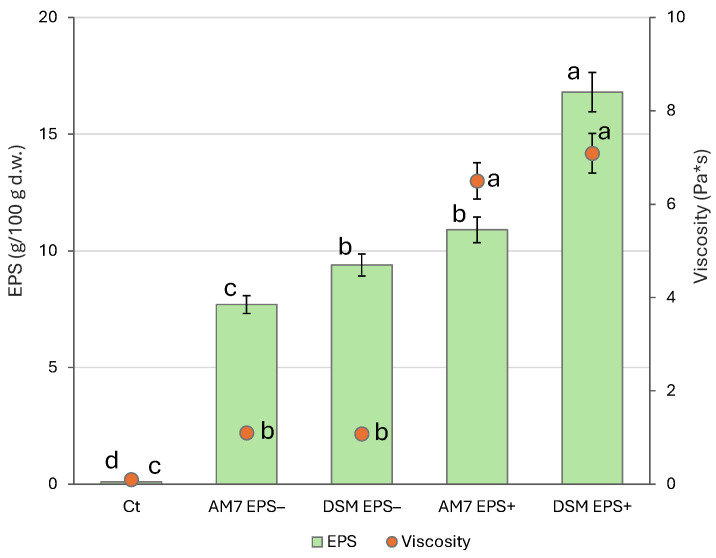
EPS concentration (g/100 g of dry weight, d.w.), and viscosity (Pa*s) of carrot-based beverages before (Ct) and after 24 h of fermentation at 25 °C with *Leuc. pseudomesenteroides* DSM20193 (DSM) and *Lv. brevis* AM7 (AM7), with (EPS^+^) or without added sucrose (EPS^−^). ^a–d^ Values with different superscript letters, within the same parameter, mean significant differences at *p* < 0.05.

Both strains confirmed the ability to produce EPS, leading to a consequent increase in viscosity (Figure 2). EPS concentration was the highest in beverages fermented with *Leuc. pseudomesenteroides* DSM20193, with up to 16.8 g/100 g of dry matter (d.m.) in DSM EPS^+^. Significantly lower values (*p* < 0.05) compared to DSM EPS^+^ were observed for AM7 EPS^+^ (10.9 ± 0.07 g/100 g d.m.). For both strains, significantly lower EPS concentration was found in beverages obtained without sucrose addition (AM7 EPS^−^ and DSM EPS^−^) compared to EPS^+^ samples.

The viscosity of the beverages was measured using a rotational viscometer before and after fermentation. In sucrose-supplemented samples, the increase in viscosity was more pronounced, reaching up to 7 Pa*s without showing any difference (*p* > 0.05) between DSM EPS^+^ and AM7 EPS^+^, and on average, 6-fold higher than EPS^−^ samples.

### 3.5. Sensory and Colour Analysis

**Figure 3:**
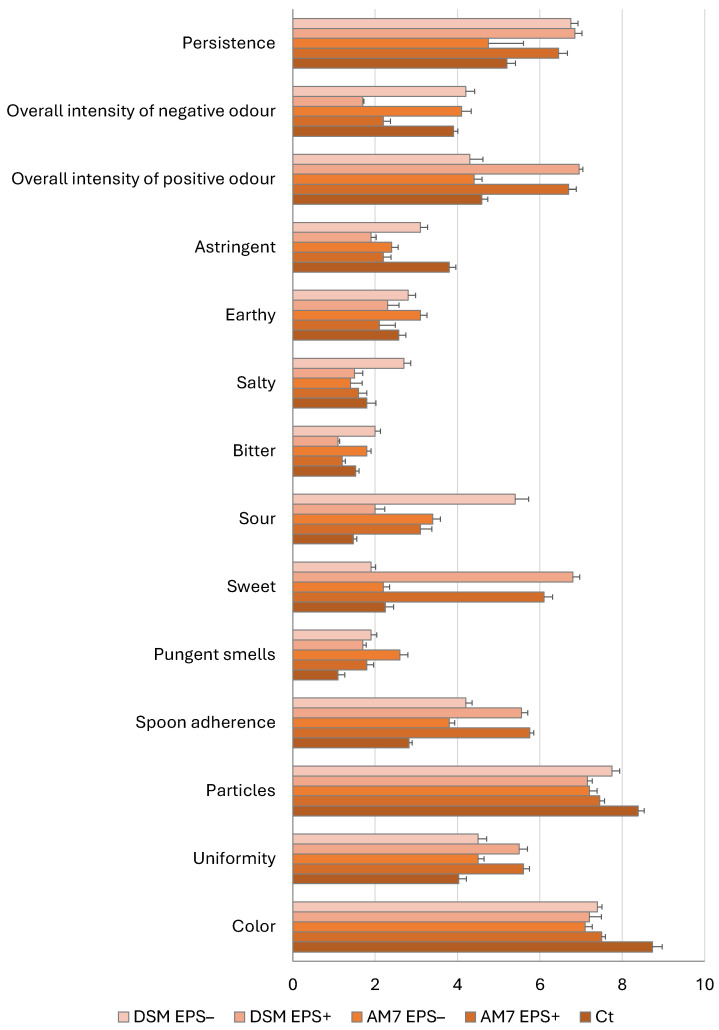
Sensory analysis of carrot-based beverages before (Ct) and after 24 h of fermentation at 25 °C with *Leuc. pseudomesenteroides* DSM20193 (DSM) and *Lv. brevis* AM7 (AM7), with (EPS^+^) or without added sucrose (EPS^−^).

The descriptive sensory analysis of the carrot-based beverages fermented with *Lv. brevis* AM7 and *Leuc. pseudomesenteroides* DSM20193, with or without added sucrose, revealed distinct differences in appearance, aroma, taste and overall perception (Figure 3). All samples exhibited high colour scores, ranging from 7.1 to 7.5 points, with a bright orange hue, particularly intense in fermented samples. Indeed, the analysis of the chromatic coordinates highlighted differences between the beverages before and after fermentation. Whereas lightness (*L*) and the blue index (*b*) were similar (*p* > 0.05) for all samples (on average 44.3 and 11.7, respectively), the red index (*a*) was significantly lower in fermented beverages (mean value −2.93) compared to Ct (−1.78 ± 0.15).

Overall, EPS^+^ beverages showed greater uniformity and spoon adherence than their EPS^−^ counterparts, and most of all, compared to the control. In terms of olfactory and taste descriptors, EPS^+^ samples were perceived as sweeter and more pleasant in general. Conversely, sourness, barely detected in Ct, was more intense in EPS^−^ beverages. Bitterness, saltiness, earthiness and astringency remained low to moderate across all samples, with slightly higher earthy and astringent notes in EPS^−^ beverages. Pungent smells and negative odour intensity were minimal in EPS^+^ beverages (ranging from 1.7 to 2.2 points) but more pronounced in EPS^−^ samples (from 4.1 to 4.2 points), whereas the overall intensity of positive odour was higher in EPS^+^ samples (on average 6.8 points) than in EPS^−^ ones (on average 4.4 points). Similarly, persistence, reflecting the duration of aftertaste, was greater in DSM EPS^+^ and AM7 EPS^+^ than in their non-sucrose controls (Figure 3).

## 4. Discussion

The global expansion of fruit and vegetable processing has generated substantial quantities of side-streams and residues that are increasingly recognised as both an environmental liability and a potential source of valuable compounds [2]. Among these, carrot residue, generated in large volumes by the juice processing sector, represents a significant underutilised by-product containing valuable dietary fibre, carotenoids and phenolic compounds [2,3]. Thus, repurposing strategies that involve its inclusion in innovative functional foods should be sought.

Based on the above considerations, the ability of two EPS-producing strains (*Leuc. pseudomesenteroides* DSM 20193 and *Lv. brevis* AM7) to synthesize EPS in carrot pomace-based beverages was evaluated. Sucrose was added to promote EPS synthesis in EPS^+^ beverages, whereas EPS^−^ samples were fermented without sucrose addition, to discriminate a possible EPS synthesis using the sucrose naturally contained in the carrot residue. All beverages, including those added to sucrose, contained more than 3 g of dietary fibre per 100 kcal of product and thus could be defined as “high in fibre”, according to EC Regulation No. 1924/2006. Typically, the quantity of sucrose employed to promote in situ EPS synthesis ranges from 2 to 20% [18,24,25]. In this study, 5% was chosen as it represented the optimal compromise between viscosity reached and residual sugar content according to preliminary tests.

During fermentation, regardless of the sucrose addition, both strains were able to grow by roughly two log cycles, reaching levels consistent with those observed in other fermented plant-based beverages [17,26]. Moreover, from a microbiological safety perspective, all fermented beverages showed *Enterobacteriaceae* counts below the detection limit (<10 cfu/mL), confirming that LAB-driven acidification effectively suppressed potentially undesirable microbial populations [27]. Acidification, reflected by the decrease in pH and increase in titratable acidity, was primarily driven by lactic and acetic acid accumulation. Overall, the ratio between lactic and acetic acids was similar for all samples except AM7 EPS^−^, which had the lowest acetic acid content. A similar trend was observed when *Lv. brevis* AM7 was used as a starter for fermentation of an oat/hemp-based yoghurt alternative, hypothesizing that the sugars present were mostly used to synthesize EPS [28]. Nevertheless, the low acetic acid content could be considered a positive trait, since it can lead to pungent odours, and an intense acidification is generally perceived as a negative trait by consumers of vegetable drinks [29]. Thus, both strains were effective acidifiers capable of stabilizing the beverage microbiologically while maintaining mild acidity suitable for consumer acceptance.

Sucrose metabolism played a pivotal role in EPS production. The nearly complete utilization of supplemented sucrose in EPS^+^ formulations suggests that both LAB strains used it as a primary carbon source for polysaccharide biosynthesis (Figure 2). Indeed, at the end of the 24 h of fermentation, the beverages contained approx. 1% of sucrose, less than 20% of the initial content (Table 3). In beverages fermented with *Leuc. pseudomesenteroides* DSM20193, either with or without sucrose addition, an increase in fructose compared to the other samples was observed. The increase in fructose is consistent with the activity of dextransucrase or glucansucrase, which cleave sucrose into glucose which is then polymerized into dextran and free fructose is released [30,31]. In contrast, in EPS^+^ AM7, fructose decreased by 60% compared to Ct. It is possible that *Lv. brevis* may have metabolised it in pathways linked to EPS biosynthesis, such as via fructokinase (responsible for the phosphorylation of fructose to fructose-6-phosphate) or the conversion to mannose-6-phosphate by mannose-6P isomerase, as proposed for other lactic acid bacteria strains [32].

Overall, EPS production can occur in the absence of sucrose addition, provided that the substrate used for fermentation exhibits an ideal monosaccharide composition conducive to their synthesis. Indeed, the carrot pomace used in this study contained approximately 15 g of sucrose per 100 g of dry matter, explaining why the fermentation of beverages with both strains resulted in EPS synthesis, even in the absence of sucrose addition (DSM EPS^−^ and AM7 EPS^−^). However, the amount was significantly lower (*p* < 0.05) than the respective beverages fermented with sucrose supplementation (Figure 2), and most notably, the viscosity was almost equivalent to that of the control beverage (ca. 6 times lower in DSM EPS^−^ and AM7 EPS^−^ compared to DSM EPS^+^ and AM7 EPS^+^). This finding suggests that the synthesised EPS may possess a distinct conformation, which consequently leads to divergent structural characteristics. Indeed, it has been recently demonstrated [25] that varying sucrose concentrations induce structural modifications of EPS, particularly alterations in branching patterns and molecular composition. These modifications, in turn, affect the techno-functional properties of the EPS.

In sucrose-supplemented beverages, despite *Lv. brevis* AM7 produced a lower amount (*p* < 0.05) of exopolysaccharides compared to *Leuc. pseudomesenteroides* DSM20193, the viscosity reached was similar (Figure 2). This is most likely due to compositional and molecular differences in the produced polymers. Indeed, *Leuc. pseudomesenteroides* DSM20193 synthesizes dextran, a homopolysaccharide, a branched polymer of D-glucose molecules bound together by a α-1-6-glycosidic bond. When the same strain was used for the fermentation of chickpea, NMR analysis found that the dextran produced had a high molecular weight and a low degree of branching, a characteristic known to induce high viscosity and improve product structure [33]. EPS produced by *Lv. brevis* AM7 was only recently studied by Wang et al. [28], who highlighted its ability to synthesize heteropolysaccharides or polysaccharides consisting of different monosaccharide units of rhamnose, mannose, glucose and glucosamine in a ratio of 10.3:12.4:58.2:19.2. These polysaccharides were also able to counteract the viscosity loss and inhibit syneresis of plant-based yoghurt alternatives during refrigerated storage [28].

Although the amount of EPS quantified might appear high, it is important to emphasize that this value is expressed on dry matter of the beverage, not on the wet product. Given that approximately 100 g of dry matter correspond to 1.4 L of fermented beverage, and considering the high water content of the system, the reported EPS concentration therefore corresponds to roughly 1.2 g per 100 mL in DSM EPS^+^, which falls within the range reported for EPS-producing strains in sucrose-supplemented plant-based fermentations [18,24].

Hence, despite the qualitative and quantitative differences, EPS synthesized from sucrose by both strains in this study were able to reduce syneresis compared to the EPS^−^ beverages, as well as confer greater adherence to the spoon during sensory analysis. These findings align with previous reports linking in situ EPS formation to improved creaminess and flavour balance in plant-based yoghurts and drinks [29,34]. Overall, the sucrose residue positively influenced sensory perception, enhancing sweetness, aroma, and textural attributes while reducing undesirable odours and excessive sourness. Indeed, EPS^+^ samples were more appreciated than EPS^−^ samples for their organoleptic features and also compared to the control beverage, which had a flatter flavour profile (Figure 3). Although the sensory analysis was not designed for multivariate or correlation-based modelling, a clear dose-dependent trend was observed across formulations. EPS^+^ beverages, which contained 3–6-fold higher EPS levels and approximately 6-fold higher viscosity than EPS^−^ samples, consistently received higher scores for spoon adherence, uniformity, persistence, and overall positive odour intensity, while simultaneously showing lower scores for negative odour intensity and sourness. These trends were observed for both strains, despite differences in absolute EPS concentration, indicating that viscosity rather than EPS alone may be the dominant driver of perceived textural improvement.

The EPS produced by several LAB species have also been shown to have a great potential as natural antioxidants [35]. As a matter of fact, the EPS-enriched beverages developed in this study showed higher DPPH radical scavenging potential than Ct and EPS^−^-samples. However, it is also likely, as shown by the significant increase (*p* < 0.05) of TPC, that the fermentation process influenced the bioaccessibility of phenolic compounds. Studies on carrot phenolic compounds and their antioxidant properties highlighted that it contains hydroxycinnamic acids (mainly chlorogenic acid) and their derivatives [7]. Indeed, LAB species can metabolize hydroxycinnamic acids through two paths (decarboxylation or reduction), producing derivatives that exert higher biological activities than their precursors [36]. Moreover, it could also be argued that the increase in TPC observed in fermented beverages (Table 3) is due to the release of bound phenolic compounds, of which carrot residue dietary fibre is extremely rich [37], thus contributing to the improved antioxidant potential. Indeed, polyphenols constitute the predominant natural antioxidants in foodstuffs. Nevertheless, they frequently occur bound to the cell wall, glycosylated, or in polymeric forms, affecting their bioaccessibility. Yet, several LAB metabolic activities, involving tannases, glucosidases, decarboxylases, reductases and esterases, are implicated in the release or conversion of these compounds into more active forms [36].

Based on the results obtained, from a sustainability standpoint, the fermentation process developed here exemplifies a practical valorisation strategy for carrot processing residues. Converting a nutrient-rich by-product into a stable, high-fibre and sensorially appealing beverage reduces waste and promotes circular bioeconomy principles [38]. The approach is scalable and cost-effective. Beyond texture and flavour enhancement, the inclusion of EPS-producing LAB may enable the formulation of clean-label and sensory appealing beverages with technologically functional and hypothesized prebiotic potential.

## 5. Conclusions

To conclude, this study demonstrated that carrot by-product is a suitable substrate for LAB fermentation aimed at in situ EPS enrichment. Both strains, *Leuc. pseudomesenteroides* DSM20193 and *Lv. brevis* AM7, effectively fermented the matrix, with sucrose addition promoting EPS synthesis, improving viscosity, and enhancing sensory acceptance. The developed beverages integrate technological feasibility, nutritional enhancement, and waste valorisation, key pillars of sustainable food innovation. 

Overall, the novelty of this study lies primarily in the repurposing strategy, which considers carob pomace as a whole matrix rather than a minor ingredient added to an entirely different product. Secondly, the fermentation process is noteworthy for its ability to facilitate in situ EPS synthesis, an essential aspect for ensuring the uniformity and complexity of the sensory profile of the beverage.

The fermentation process described is technically feasible for industrial scale-up, as it relies on well-established food fermentation principles and industrially compatible lactic acid bacteria that have QPS (quality presumption of safety) status. However, challenges related to raw material variability and consumer acceptance may occur. Hence, further pilot-scale studies will be required to optimize process parameters as well as validate product stability and functionality under industrial conditions. Moreover, future studies could focus on the assessment of the in vitro prebiotic activity and ex vivo and in vivo antioxidant activity of the beverages, to further validate the results observed so far.

## Figures and Tables

**Table 1:** Formulation of carrot-based beverages. Ct, non- fermented beverage; AM7 EPS^−^ and AM7 EPS^+^, beverages fermented with *Lv. brevis* AM7; DSM EPS^−^ and DSM EPS^+^, beverages fermented with *Leuc. pseudomesenteroides* DSM20193.

	Ct	AM7 EPS^−^	DSM EPS^−^	AM7 EPS^+^	DSM EPS^+^
**Carrot pomace (g)**	7.5	7.5	7.5	7.5	7.5
**Sucrose (g)**	-	-	-	15	15
**Water (mL)**	292.5	292.5	292.5	277.5	277.5
**LAB inoculum (log cfu/mL)**	-	6.5	6.5	6.5	6.5

**Table 2:** Proximal composition (expressed as g/100 g) of carrot-based beverages before (Ct) and after 24 h of fermentation at 25 °C with *Leuc. pseudomesenteroides* DSM20193 (DSM) and *Lv. brevis* AM7 (AM7), with (EPS^+^) or without added sucrose (EPS^−^).

	Ct	AM7 EPS^−^	DSM EPS^−^	AM7 EPS^+^	DSM EPS^+^
**Total carbohydrates**	2.13 ± 0.07 ^b^	2.05 ± 0.06 ^b^	2.04 ± 0.10 ^b^	3.06 ± 0.15 ^a^	3.05 ± 0.8 ^a^
of which sugars	0.60 ± 0.05 ^b^	0.54 ± 0.02 ^b^	0.51 ± 0.04 ^a^	1.62 ± 0.07 ^a^	1.59 ± 0.01 ^a^
of which fibres	1.53 ± 0.03 ^a^	1.51 ± 0.05 ^a^	1.53 ± 0.05 ^a^	1.44 ± 0.07 ^a^	1.46 ± 0.06 ^a^
**Proteins**	0.17 ± 0.02 ^a^	0.15 ± 0.00 ^a^	0.15 ± 0.00 ^a^	0.14 ± 0.01 ^a^	0.14 ± 0.01 ^a^
**Fats**	0.04 ± 0.01 ^a^	0.04 ± 0.00 ^a^	0.04 ± 0.00 ^a^	0.03 ± 0.01 ^a^	0.03 ± 0.00 ^a^
**Ashes**	0.12 ± 0.01 ^a^	0.11 ± 0.00 ^a^	0.12 ± 0.00 ^a^	0.10 ± 0.01 ^a^	0.10 ± 0.01 ^a^
**Energy value (kcal/kJ)**	6.6/27.2 ^b^	7.4/31.5 ^b^	6.2/25.4 ^b^	10.2/42.6 ^a^	10.1/42.3 ^a^

^a,b^ Values in the same row with different superscript letters mean significant differences at *p* < 0.05.

**Table 3:** Main biochemical and nutritional features of carrot-based beverages before (Ct) and after 24 h of fermentation at 25 °C with *Leuc. pseudomesenteroides* DSM20193 (DSM) and *Lv. brevis* AM7 (AM7), with (EPS^+^) or without added sucrose (EPS^−^).

	Ct	AM7 EPS^−^	DSM EPS^−^	AM7 EPS^+^	DSM EPS^+^
**pH**	5.87 ± 0.04 ^a^	4.80 ± 0.06 ^b^	4.70 ± 0.07 ^b^	4.67 ± 0.05 ^b^	4.70 ± 0.07 ^b^
**TTA (mL)**	0.7 ± 0.06 ^c^	1.4 ± 0.21 ^ab^	1.0 ± 0.27 ^b^	1.5 ± 0.28 ^a^	1.1 ± 0.12 ^ab^
**Glucose (g/L)**	0.46 ± 0.00 ^a^	0.27 ± 0.01 ^b^	0.28 ± 0.04 ^ab^	0.03 ± 0.02 ^c^	0.23 ± 0.02 ^b^
**Fructose (g/L)**	0.96 ± 0.00 ^c^	0.93 ± 0.01 ^c^	1.27 ± 0.02 ^b^	0.62 ± 0.02 ^d^	1.60 ± 0.03 ^a^
**Sucrose (g/L)**	4.63 ± 0.01 ^b^	3.14 ± 0.04 ^c^	2.58 ± 0.09 ^d^	11.43 ± 0.02 ^a^	11.25 ± 0.20 ^a^
**Lactic acid (mmol/L)**	0.28 ± 0.17 ^c^	3.34 ± 0.04 ^a^	2.55 ± 0.10 ^b^	2.27 ± 0.84 ^b^	2.72 ± 0.00 ^b^
**Acetic acid (mmol/L)**	0.00 ± 0.00 ^d^	0.84 ± 0.18 ^ab^	1.05 ± 0.00 ^a^	0.11 ± 0.11 ^c^	1.05 ± 0.29 ^a^
**TPC (mg GAE eq/g d.m.)**	68.5 ± 2.51 ^c^	77.7 ± 4.08 ^b^	90.5 ± 26.0 ^a^	113.6 ± 8.46 ^a^	107.4 ± 18.50 ^a^
**DPPH radical scavenging activity (%)**	54.3 ± 1.12 ^c^	71.9 ± 6.16 ^b^	78.2 ± 3.1 ^b^	83.4 ± 3.61 ^a^	84.6 ± 1.87 ^a^

^a–d^ Values in the same row with different superscript letters mean significant differences at *p* < 0.05.

## Data Availability

The contributions presented in this study are included in the article. Further inquiries can be directed to the corresponding author.

## Abbreviations

The following abbreviations are used in this manuscript:

DPPH	1,1-diphenyl-2-picrylhydrazyl
EPS	exopolysaccharides
LAB	lactic acid bacteria
MRS	De Man, Rogosa and Sharp
QPS	quality presumption of safety
TPC	total polyphenol content
TTA	total titratable acidity
